# Does the Adequacy Parameter Kt/V_urea_ Reflect Uremic Toxin Concentrations in Hemodialysis Patients?

**DOI:** 10.1371/journal.pone.0076838

**Published:** 2013-11-13

**Authors:** Sunny Eloot, Wim Van Biesen, Griet Glorieux, Nathalie Neirynck, Annemieke Dhondt, Raymond Vanholder

**Affiliations:** Nephrology Section, Department of Internal Medicine, Ghent University Hospital, Gent, Belgium; University of Florida, United States of America

## Abstract

Hemodialysis aims at removing uremic toxins thus decreasing their concentrations. The present study investigated whether Kt/V_urea_, used as marker of dialysis adequacy, is correlated with these concentrations. Predialysis blood samples were taken before a midweek session in 71 chronic HD patients. Samples were analyzed by colorimetry, HPLC, or ELISA for a broad range of uremic solutes. Solute concentrations were divided into four groups according to quartiles of Kt/V_urea_, and also of different other parameters with potential impact, such as age, body weight (BW), Protein equivalent of Nitrogen Appearance (PNA), Residual Renal Function (RRF), and dialysis vintage. Dichotomic concentration comparisons were performed for gender and Diabetes Mellitus (DM). Analysis of Variance in quartiles of Kt/V_urea_ did not show significant differences for any of the solute concentrations. For PNA, however, concentrations showed significant differences for urea (P<0.001), uric acid (UA), p-cresylsulfate (PCS), and free PCS (all P<0.01), and for creatinine (Crea) and hippuric acid (HA) (both P<0.05). For RRF, concentrations varied for β_2_-microglobulin (P<0.001), HA, free HA, free indoxyl sulfate, and free indole acetic acid (all P<0.01), and for p-cresylglucuronide (PCG), 3-carboxy-4-methyl-5-propyl-2-furanpropionic acid (CMPF), free PCS, and free PCG (all P<0.05). Gender and body weight only showed differences for Crea and UA, while age, vintage, and diabetes mellitus only showed differences for one solute concentration (UA, UA, and free PCS, respectively). Multifactor analyses indicated a predominant association of concentration with protein intake and residual renal function. In conclusion, predialysis concentrations of uremic toxins seem to be dependent on protein equivalent of nitrogen appearance and residual renal function, and not on dialysis adequacy as assessed by Kt/V_urea_. Efforts to control intestinal load of uremic toxin precursors by dietary or other interventions, and preserving RRF seem important approaches to decrease uremic solute concentration and by extension their toxicity.

## Introduction

Failure of the kidneys is associated with the gradual retention of a myriad of solutes [1], causing an endogenous intoxication and failure of almost all organ systems [2], [3]. Dialysis aims at removing those solutes, resulting in a decrease of their concentration and hence also their biological toxicity.

Quantification of the removal of these toxins may offer a useful tool to evaluate the adequacy of a given approach, but might also lead to misconceptions, e.g. by abandoning attempts to further improve a removal strategy once a set target is reached. Traditionally, Kt/V_urea_ is the marker of dialysis adequacy that is most widely used on a regular basis. In some countries, reaching a threshold Kt/V_urea_ is even a prerequisite for reimbursement of dialysis [4].

When considering observational data, one cannot deny that the introduction of Kt/V_urea_ in 1985 helped to gradually improve survival of the dialysis population [5]–[8]. The parameter was however developed in an era when dialysis was almost exclusively performed over relatively short sessions with small pore dialyzers [9]. Many strategic modifications have meanwhile been introduced, such as large pore membranes, convection, frequent dialysis and extended dialysis. All these alternative strategies enhance solute removal [10]–[16] and have been associated with improved outcome [15], [17]–[23], but do not necessarily increase Kt/V_urea_ [10], [11], [22], [23]. Also the number of known uremic toxins has extended [24], [25], mostly with compounds that are difficult to remove by standard dialysis.

In two recent studies, we demonstrated in CKD patients not yet on dialysis that estimated GFR (eGFR), as the main currently used marker of renal function, was barely and inconsistently associated with concentrations of uremic toxins, although the latter are associated with organ dysfunction [26], [27]. This divergence was attributed to a greater impact on uremic solute concentration by factors other than GFR, such as diet, intestinal generation, metabolism and tubular secretion [28].

Thus, the question could be raised in how far this would be true as well for the main marker of dialysis adequacy Kt/V_urea_. Although Kt/V_urea_ is mathematically related to the concentration change during dialysis, one could assume that keeping this parameter at a higher threshold would result in a decrease of uremic solute concentration as a consequence of more efficient removal, also before dialysis.

An additional asset of performing this type of study in hemodialysis, is that in this setting, it is quite easy to assess objectively dietary protein intake, one of the potentially impacting factors [29]. Remarkably enough, this question has to the best of our knowledge never been studied, although a recent review pointed to the theoretical possibility that Kt/V_urea_ would not be an accurate marker for uremic solute removal and retention [21].

Therefore, in this study we want to clarify: 1/whether Kt/V_urea_ is representative for the concentration of a broad array of uremic toxins in patients on hemodialysis; 2/whether there is a difference in uremic toxin concentrations depending on other patient characteristics such as age, body weight, protein equivalent of nitrogen appearance, residual renal function, dialysis vintage, gender, and diabetes mellitus.

## Patients and Methods

### Ethics Statement

The study was approved by the local Ethics Committee (Ethical Committee, Ghent University Hospital, Ghent, Belgium) and performed in accordance to the Declaration of Helsinki. Written informed consent was obtained from all participants.

### Patients

All chronic hemodialysis patients, older than 18 years, with a residual renal function lower than 10 mL/min, and dialyzed in our centre during the day, were considered for inclusion. Patients were on maintenance hemodialysis for at least three months during which the same hemodialyzer, dialysis duration, blood and dialysate flows, convective strategy, and vascular access had been applied. Dialysis dose, expressed as Kt/V_urea_ is calculated monthly according to the single-pool Daugirdas formula [30] to check accordance to the EBPG [31], and is only adapted if the value goes below threshold. The general exclusion criteria were active infection, pregnancy, unstable condition, and vascular access problems.

### Sampling and data collection

Predialysis blood samples were taken during a midweek session, immediately centrifuged (3000 rpm corresponding to 1250 g), aliquoted, and stored at −80°C until analysis. Postdialysis blood samples, as needed for the calculation of Kt/V_urea_ and PNA, were taken immediately after discontinuation of the dialysis session after slowing the blood pump to 100 mL/min during 15 s.

Urea [molecular weight MW: 60D] and creatinine (Crea) [MW: 113D] were measured by standard laboratory methods.

Different solutes, most of them protein bound, were determined by high performance liquid chromatography (HPLC): uric acid (UA) [168D], hippuric acid (HA) [179D, protein binding (PB)∼50%], 3-carboxy-4-methyl-5-propyl-2-furanpropionic acid (CMPF) [240D - PB∼100%], indoxyl sulfate (IS) [213D - PB∼90%], indole acetic acid (IAA) [175D - PB∼65%], *p*-cresylsulfate (PCS) [187D - PB∼95%], and *p*-cresylglucuronide (PCG) [284D - PB∼10%]. To determine the total fraction, serum samples were first deproteinized by heat denaturation [32] and analyses were performed by reverse-phase HPLC. IS and IAA (excitation λ_ex_:280 nm; emission λ_em_:340 nm) and PCS and PCG (λ_ex_:265 nm; λ_em_:290 nm) were determined by fluorescence analysis, and HA and CMPF were analyzed by UV detection at 254 nm [14]. Free fractions were determined according to Fagugli et al [14].

ELISA kits manufactured by DLD Diagnostika GmbH (Hamburg, Germany) were used for measuring asymmetric dimethylarginine (ADMA) [202D], symmetric dimethylarginine (SDMA) [202D] after acylation. For beta-2-microglobulin (β_2_M) [11.8 kD], ELISA kits of Orgentec Diagnostika GmbH (Mainz, Germany) were used.

For each patient, the following additional parameters were registered: age, gender, body weight (BW), presence of diabetes mellitus (DM), dialysis vintage, and medication, while Kt/V_urea_, residual renal function (RRF), and protein equivalent of nitrogen appearance (PNA) were calculated as explained below.

### Calculations

Since the single treatment dose as measured during a midweek dialysis session does not reflect the average clearance both by exogenous hemodialysis and endogenous residual renal clearance, we calculated weekly Kt/V_urea_ accounting for ultrafiltration and residual renal function according to Daugirdas et al [33]:
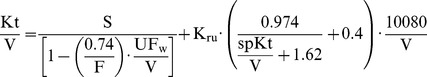
(1)with F the number of dialysis sessions per week, K_ru_ the residual renal urea clearance (mL/min), UF_w_ the weekly fluid removal (L), V the urea distribution volume (L) as calculated according to Daugirdas and Smye [34], and S the standard Kt/V according to Leypoldt:
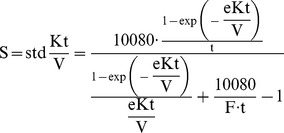
(2)with t the treatment time (min), and eKt/V calculated according to [35]:
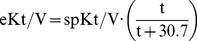
(3)And spKt/V calculated according to Daugirdas et al [30]:
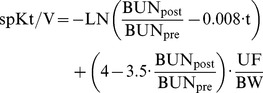
(4)with BUN_pre_ and BUN_post_ the pre and post dialysis Blood Urea Nitrogen concentration, UF the ultrafiltration volume, and BW the post dialysis body weight.

Residual Renal Function (RRF) was calculated as the arithmetic mean of the creatinine and urea clearance (Cl_Crea_ and Cl_urea_, respectively):
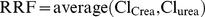
(5)with:
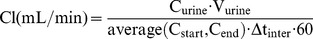
(6)with C_urine_ and V_urine_ (mL) the concentration and volume of the urine as collected during the interdialytic period Δt_inter_ (h), and C_start_ and C_end_ the blood concentrations at the start and end of the interdialytic period. Herewith, C_start_ was corrected for the post-dialysis rebound as [36]:
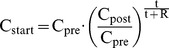
(7)With C_pre_ and C_post_ the pre and immediate post-dialysis concentrations, and R the rebound time, i.e. 35 min for urea and 70 min for Crea.

Finally, the Protein equivalent of Nitrogen Appearance (PNA) at midweek was calculated according to the K/DOQI and EBPG [37], [38]:
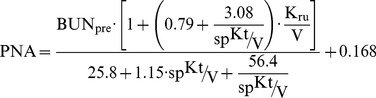
(8)


### Statistical analyses

For the patient characteristics age, BW, Kt/V_urea_, PNA, and dialysis vintage, the median and 25^th^ and 75^th^ percentile were calculated and, per characteristic, patients were divided into four groups according to quartiles. Since the median of RRF was zero, the four groups for this parameter were not defined by quartiles but consisted of one group to which all anuric patients were selected (n = 40), whereas the remaining non-anuric patients were distributed in tertiles (n = 10–11 each). For gender and DM, patients were divided in two groups according to male/female and DM/non DM. With these data, non-parametric analysis of variance (Kruskal-Wallis) tests were performed to check potential differences in uremic toxin concentrations among the groups.

Furthermore, correlations (Spearman) were evaluated between uremic toxin concentrations and the different studied characteristics; finally, multifactor analyses per solute were performed.

The data of patients taking allopurinol were excluded from the analysis considering uric acid.

Data are expressed as mean ± standard deviation for normal distributions, or as medians with the 25^th^ and 75^th^ percentiles for non normal distributions.

P<0.05 was considered to be statistically significant. All statistical analyses were performed using SPSS Statistics 19 (SPSS Inc, Chicago, IL) for Windows (Microsoft Corp, Redmond, WA).

## Results

Seventy-four chronic hemodialysis patients fulfilled the inclusion criteria. Two of them declined written consent, and 1 patient had an interdialytic interval of 2.5 days at midweek instead of 1.5 days. The 71 chronic hemodialysis patients included were on thrice weekly hemodialysis for 38.0 (19.5–61.5) months: 26/45 females/males, 28 diabetes mellitus, 31 with RRF, age 72 (63.0–80.0) years, body weight 69.0 (61.3–78.8) kg, weekly Kt/V_urea_ 2.43 (2.28–2.63), and PNA 0.83 (0.74–1.01) g/kg/day (Table 1).

**Table 1:** Patient characteristics and Kt/V values.

	Median (25^th^–75^th^ percentile)*
Gender (female/male)	26/45
DM (yes/no)	28/43
RRF (yes/no)	31/40
Allopurinol (yes/no)	7/64
Age (years)	72.0 (63.0–80.0)
BW (kg)	69.0 (61.3–78.8)
spKt/V	1.62 (1.45–1.89)
eKt/V	1.43 (1.28–1.66)
weekly Kt/V	2.43 (2.28–2.63)
PNA (g/kg/day)	0.83 (0.74–1.01)
Dialysis vintage (months)	38.0 (19.5–61.5)

* if applicable.

DM: diabetes mellitus, BW: body weight, RRF: residual renal function, PNA: protein equivalent of nitrogen appearance.

Our patients were routinely dialyzed for 245±18 min, with a blood flow of 321±37 mL/min (Q_B_ range 220–350 mL/min) and Q_D_ = 500 mL/min in hemodialysis mode with either an FX8 (Fresenius Medical Care, Germany) (n = 5), or Evodial (Gambro, Sweden) (n = 1) dialyzer, or in postdilution hemodiafiltration mode with either an FX800 (Fresenius Medical Care, Germany) (n = 41), Phylter 17SD (Bellco, Italy) (n = 7), Xenium 210 (Baxter, USA) (n = 6), PHF (Bellco, Italy) (n = 4), Polyflux 170H (Gambro, Sweden) (n = 4), or Surflux 170UH (Nipro, Japan) (n = 3) dialyzer. All patients had a permanent vascular access: i.e. 42 fistulas and 29 tunneled central venous catheters.

An overview of the measured predialysis concentrations of uremic solutes is given in Table 2. The percentage differences between minimum and maximum concentrations among the patients ranged from 72.0 (UA) to 99.5% (total and free PCG) (Table 2); normalizing concentrations to Kt/V_urea_ resulted in the same percentage differences between the minimum and maximum values (data not shown).

**Table 2:** Uremic toxin concentrations.

	median (25^th^–75^th^p)	[(75^th^-25^th^)/75^th^]·100	min - max	[(max-min)/max]·100
urea	0.90 (0.71–1.06)	33.5	0.18–1.88	90.4
Crea	7.19 (5.77–9.01)	36.0	3.35–14.9	80.4
ADMA	1.00 (0.84–1.18)	28.2	0.51–1.93	73.6
SDMA	2.08 (1.73–2.48)	30.3	0.77–3.79	79.7
UA	6.53 (5.62–7.06)	20.3	2.55–9.11	72.0
β_2_M	39.4 (28.5–48.1)	40.8	13.9–96.3	89.6
HA	2.94 (1.47–5.64)	74.0	0.24–14.21	98.3
IS	1.80 (1.27–2.77)	54.4	0.29–5.75	97.7
IAA	0.18 (0.13–0.23)	42.7	0.07–0.73	90.9
PCS	2.94 (1.73–4.36)	60.2	0.06–7.53	99.2
PCG	0.49 (0.18–0.89)	79.8	0.02–3.94	99.5
CMPF	0.38 (0.20–0.61)	68.0	0.06–1.37	95.8
Free HA	1.65 (0.70–3.32)	79.0	0.13–9.09	98.6
Free IS	0.11 (0.06–0.23)	72.4	0.02–0.72	97.8
Free IAA	0.06 (0.04–0.07)	48.1	0.02–0.22	91.2
Free PCS	0.18 (0.11–0.33)	66.4	0.04–0.66	94.7
Free PCG	0.48 (0.17–0.86)	80.1	0.02–3.59	99.5

mg/dL, except urea in g/L, β_2_M in µg/mL, and ADMA and SDMA in µmol/L.

Crea: creatinine, ADMA: asymmetric dimethylarginine, SDMA: symmetric dimethylarginine, UA: uric acid, β_2_M: beta-2-microglobulin, HA: hippuric acid, IS: indoxyl sulfate, IAA: indole acetic acid, PCS: *p*-cresylsulfate, PCG: *p*-cresylglucuronide, CMPF: 3-carboxy-4-methyl-5-propyl-2-furanpropionic acid.

**Figure 1:**
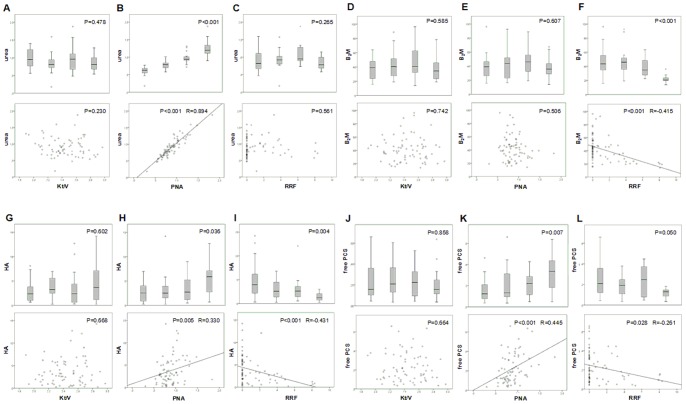
Box plots of quartiles and correlations of different solute concentrations versus Kt/V, PNA, and RRF: urea in g/L (panels A, B, and C), β_2_M in µg/mL (panels D, E, and F), HA in mg/dL (panels G, H, and I), and free PCS in mg/dL (panels J, K, and L). β_2_M: beta-2-microglobulin, HA: hippuric acid, PCS: *p-*cresylsulfate, PNA: protein equivalent of nitrogen appearance, RRF: residual renal function.

Table 3 shows the P-values of the Kruskal-Wallis tests for the concentrations under study if patient characteristics were separated in quartiles or dichotomically. The main characteristics associated with a significant difference of solute concentrations were PNA and RRF (Table 3 and Figure 1). For Kt/V_urea_ no single significant difference was found.

**Table 3:** P-values of comparison between concentrations for characteristics as divided in quartiles or dichotomically.

	Age	BW	Kt/V	PNA	RRF	vintage	gender	DM
urea	0.097	0.116	0.478	**<0.001**	0.265	0.219	0.310	0.728
Crea	0.129	**0.015**	0.339	**0.027**	0.345	0.441	**0.004**	0.842
ADMA	0.616	0.458	0.396	0.848	0.337	0.683	0.492	0.814
SDMA	0.408	0.891	0.297	0.548	0.196	0.794	0.797	0.668
UA	**0.002**	**0.026**	0.097	**0.004**	0.240	**0.026**	**0.025**	0.171
β_2_M	0.898	0.666	0.585	0.607	**<0.001**	0.644	0.346	0.916
HA	0.539	0.577	0.602	**0.036**	**0.004**	0.268	0.247	0.378
IS	0.850	0.462	0.340	0.261	0.159	0.664	0.839	0.259
IAA	0.678	0.251	0.583	0.203	0.089	0.959	0.437	0.402
PCS	0.283	0.698	0.809	**0.001**	0.815	0.739	0.452	0.312
PCG	0.230	0.786	0.391	0.067	**0.041**	0.766	0.273	0.291
CMPF	0.065	0.537	0.316	0.464	0.073	0.984	0.865	0.106
Free HA	0.491	0.580	0.629	0.092	**0.002**	0.181	0.277	0.384
Free IS	0.505	0.605	0.620	0.266	**0.006**	0.772	0.976	0.110
Free IAA	0.633	0.219	0.480	0.660	**0.002**	0.978	0.440	0.107
Free PCS	0.263	0.604	0.858	**0.007**	**0.050**	0.601	0.445	**0.036**
Free PCG	0.205	0.763	0.409	0.070	**0.038**	0.777	0.300	0.317

Crea: creatinine, ADMA: asymmetric dimethylarginine, SDMA: symmetric dimethylarginine, UA: uric acid, β_2_M: beta-2-microglobulin, HA: hippuric acid, IS: indoxyl sulfate, IAA: indole acetic acid, PCS: *p*-cresylsulfate, PCG: *p*-cresylglucuronide, CMPF: 3-carboxy-4-methyl-5-propyl-2-furanpropionic acid.

BW: body weight, PNA: protein equivalent of nitrogen appearance, RRF: residual renal function, DM: diabetes mellitus.

P<0.05 is indicated in bold.

Table 4 shows the R and P-values of the Spearman correlations between solute concentrations and patient characteristics. Kt/V_urea_ showed a correlation only with the concentration of Crea. PNA, on the contrary, showed significant correlations for the majority of solutes with R = 0.894 for urea, and a value in the range 0.4–0.6 for UA, PCS, and free PCS, and 0.2–0.4 for Crea, HA, IS, PCG, free HA, and free PCG. RRF showed inversed correlations with an R-value in the range −0.4 to −0.6 for β_2_M, HA, free HA, and free IAA, and with R between −0.2 and −0.4 for IS, PCG, free IS, free PCS, and free PCG.

**Table 4:** P and R-values for Spearman correlations between the solute concentrations and the different patient characteristics.

		Age	BW	Kt/V	PNA	RRF	vintage
urea	P	**0.007**	0.511	0.230	**<0.001**	0.561	0.210
	R	**−0.320**			**0.894**		
Crea	P	**0.009**	**0.009**	**0.048**	**0.004**	0.207	0.332
	R	**−0.311**	**0.312**	**−0.237**	**0.336**		
ADMA	P	0.657	0.880	0.118	0.577	0.608	0.950
SDMA	P	0.552	0.962	0.160	0.211	0.427	0.470
UA	P	**<0.001**	**0.041**	0.058	**0.001**	0.243	**0.007**
	R	**−0.446**	**0.256**		**0.373**		**−0.336**
β_2_M	P	0.913	0.837	0.742	0.506	**<0.001**	0.305
	R					**−0.415**	
HA	P	0.962	0.490	0.668	**0.005**	**<0.001**	0.232
	R				**0.330**	**−0.431**	
IS	P	0.967	0.196	0.801	**0.046**	**0.022**	0.282
	R				**0.237**	**−0.272**	
IAA	P	0.658	0.602	0.140	0.801	0.101	0.782
PCS	P	0.456	0.941	0.712	**<0.001**	0.829	0.809
	R				**0.529**		
PCG	P	0.825	0.734	0.651	**0.003**	**0.004**	0.540
	R				**0.349**	**−0.335**	
CMPF	P	**0.003**	0.154	0.576	0.562	0.080	0.559
	R	**0.346**					
Free HA	P	0.840	0.398	0.824	**0.024**	**<0.001**	0.229
	R				**0.267**	**−0.453**	
Free IS	P	0.953	0.361	0.870	0.131	**0.001**	0.668
	R					**−0.380**	
Free IAA	P	0.964	0.451	0.084	0.525	**<0.001**	0.653
	R					**−0.410**	
Free PCS	P	0.959	0.612	0.664	**<0.001**	**0.028**	0.238
	R				**0.445**	**−0.261**	
Free PCG	P	0.792	0.746	0.639	**0.003**	**0.004**	0.536
	R				**0.346**	**−0.337**	

Crea: creatinine, ADMA: asymmetric dimethylarginine, SDMA: symmetric dimethylarginine, UA: uric acid, β_2_M: beta-2-microglobulin, HA: hippuric acid, IS: indoxyl sulfate, IAA: indole acetic acid, PCS: *p*-cresylsulfate, PCG: *p*-cresylglucuronide, CMPF: 3-carboxy-4-methyl-5-propyl-2-furanpropionic acid.

BW: body weight, PNA: protein equivalent of nitrogen appearance, RRF: residual renal function.

P<0.05 is indicated in bold. R values only shown in case of significant P.

Also in multifactor analysis, PNA and RRF were the main factors associated with uremic solute concentration, except for Crea, and this occurred mainly independently (Table 5).

**Table 5:** Multifactor analysis: covariates with cumulative R-value.

Solute	Covariates/R-value of full model	
Urea	PNA/0.894	
Crea	PNA/0.336	Kt/V/0.624
ADMA		
SDMA		
UA	PNA/0.373	
β_2_M	RRF/−0.415	
HA	RRF/−0.431	
IS	RRF/−0.272	
IAA		
PCS	PNA/0.529	
PCG	PNA/0.349	RRF/0.441
CMPF	age/0.346	
Free HA	RRF/−0.453	
Free IS	RRF/−0.380	
Free IAA	RRF/−0.410	
Free PCS	PNA/0.445	
Free PCG	PNA/0.346	RRF/0.438

Empty cells indicate that the correlation analysis did not include other elements that contributed significantly.

## Discussion

In a group of 71 hemodialysis patients, variances were studied for a broad range of predialysis uremic toxin concentrations as functions of different patients characteristics and Kt/V_urea_. The most important findings are that different quartiles of Kt/V_urea_ did not show any variations in serum concentration of the studied solutes, whereas quartiles of PNA and RRF showed significant variations of serum levels for 6 and 8 solutes under study, respectively. For correlation analysis, only one association was found for Kt/V_urea_, whereas there were 10 associations for PNA and 9 for RRF, pointing into the same direction as the analysis per quartiles.

UA, ADMA, PCS, IS, and β_2_M have repeatedly been associated with vascular damage and mortality in renal and general populations [39]–[49]. In our study, quartiles of weekly Kt/V_urea_ did not show any significant variance in serum concentration of these solutes as was also found for spKt/V and eKt/V (data not shown). The weekly Kt/V_urea_, per definition accounting for RRF, was correlated to only one investigated solute concentration, while no correlations were found when considering spKt/V or eKt/V (data not shown). On the other hand, subdivision for quartiles of PNA showed differences in the serum concentration of UA, PCS, urea, Crea, HA, and free PCS, and showed, additionally, correlations with the concentrations of IS, PCG, free HA, and free PCG. Furthermore, quartiles of RRF showed differences for β_2_M, HA, PCG, and free concentrations of HA, IS, IAA, PCS, and PCG, and showed, additionally, correlations with the IS concentrations. Hence, from the present study, Kt/V_urea_ does not seem to be a very good predictor of uremic solute concentrations, not even in the subpopulation of patients without any residual renal function (data not shown).

During the last decade, it became clear that dialysis dose, as measured by the removal of a low-molecular weight solute such as urea as is the case with Kt/V_urea_, may not entirely be a parameter of dialysis adequacy. Kinetics of urea differ from that of other uremic solutes, even when these are small and water soluble like urea is [50], [51]. Furthermore, increasing dialysis time from 4 up to 8 hours, without changing the quantity of processed blood and dialysate, did not impact Kt/V_urea_, whereas, β_2_M, a middle molecule and phoshate, a small but difficult to remove solute, showed an increase by 81 and 49% respectively [11]. The present study here adds that not only solute removal but also predialysis solute concentration is not reflected in Kt/V_urea_. At first sight, this could be an evident conclusion, since Kt/V_urea_ is mathematically related to the change in urea concentration during dialysis rather than to the absolute concentration. However, the ultimate goal of hemodialysis is to decrease absolute toxic solute concentrations, and with it, the biological activity of uremic toxins. Hence, keeping the adequacy parameter Kt/V_urea_ above the prescribed threshold should result in decreased concentrations.

Fairly similar deceiving results, pleading against a unique universal marker of uremic solute removal, were obtained in two recent studies evaluating the associations of uremic retention solute concentrations with estimated Glomerular Filtration Rates (eGFR) in patients of Chronic Kidney Disease (CKD) stage 2 to 5, not on dialysis [26], [27]. These data strongly suggest that other factors than GFR are more powerful determinants of serum solute concentration [26]. These include renal tubular secretion (IS, HA) [52], metabolic factors (ADMA) [53], intestinal secretion, absorption and generation (UA) [54], (indoles, phenols) [29], [55], [56], (IS) [57], [58].[26], [59], [60]. Impressive differences in serum concentration of different solutes, either adjusted or not for Kt/V_urea_, were found in our present study (ranging from 72 to close to 100%), confirming previous data in another hemodialyzed population (concentration differences ranging from 66 to 100%) [26], so that the same interfering factors may also play an as important role in dialysis patients as in non-dialyzed CKD patients.

We found that residual renal function (RRF) distinguishes well among solute concentrations. This confirms previous findings that RRF has a higher impact on patient outcome than a high delivered Kt/V_urea_ [61], and that RRF is better associated with optimal nutritional status than dialysis adequacy [62]. In addition, even limited increments of RRF are substantially more important than dialyzer clearance as a determinant of β_2_M concentrations in patients on high flux hemodialysis [63] and even hemodiafiltration, in spite of enhanced β_2_M clearance with this strategy due to convection [64], [65]. Also for the protein bound compounds, RRF has a major contribution to total solute removal of patients on dialysis [66], [67].

The contribution of RRF to the differences between patients in the serum concentration of β_2_M is in correspondence with the inverse associations between RRF and another middle molecule, Fibroblast Growth Factor-23 (FGF23) as previously found in a prospective observational cohort study in peritoneal and hemodialysis patients [68].

Our findings for RRF as predictor for most uremic solute concentrations in the dialysis population do not fully match our previous findings for eGFR as predictor for solute concentrations in CKD 2–5 patients not on dialysis [69]–[72]. This might be due to a difference in calculation of the renal function: i.e. while RRF in this study is assessed directly using concentrations of Crea and urea in blood and urine, in our previous studies eGFR was estimated only using blood concentrations of Crea and/or CystC [69]–[72]. In addition, GFR in the present dialysis population is much lower than in the earlier CKD stages, whereas dialysis is an additional confounder affecting concentration. Thus conditions in between studies were entirely different.

Our data with regards to residual renal function point into the same direction as those recently reported by Marquez et al [69]–[72], also showing inverse correlations between protein bound solute concentrations and residual renal function, even if they did not take into account a measure of protein intake such as PNA.

Regarding PNA, significant correlations were found with predialysis urea concentrations, as could be expected from the PNA formula (equation 8). However, PNA is also well related to substantial differences in serum concentrations of many other solutes under study. These data are consistent with growing evidence that food protein intake and its intestinal processing, to a large extent regulated by the gut microbiota, plays an important role in uremic toxin generation, especially in relation with protein bound molecules derived from amino acid metabolites such as indole or p-cresol [29], [73], [74]. Of note, whereas quartiles of PNA showed variances in concentrations of solutes that are amino acid metabolites and thus depending on protein intake, this was not the case for β_2_M which, as a component of major histocompatibility complex class I molecules, is of endogenous origin.

Our data might seemingly be in contradiction with the current understanding that inadequate nutrition is an important predictor of outcome in chronic hemodialysis patients [75]–[77], since low values of PNA have been associated with higher morbidity and mortality [78], [79] whereas here they are linked to lower uremic toxin concentrations. However, for several uremic retention solutes such as AGEs and Hcy, a low rather than high concentration has been associated with higher mortality in dialysis patients [69]–[72]; these differences have been attributed to the fact that the toxic effect of the molecules was overridden by the deleterious impact of malnutrition and the lower intake of solutes or their precursors when less food is entering the intestine [69], [71]. For protein bound toxins, like indoxyl sulfate or p-cresylsulfate, however, high, not low, concentration has repeatedly been associated with mortality, even after adjustment for protein intake parameters [39], [40], [42], suggesting that toxin concentration in this case overrides the impact of nutrition on outcome. This does not take away that low protein intake should be avoided in hemodialysis patients due to its negative impact on outcome per se [77], [78]. Our data do not refute high protein intakes but are only proof of concept that the intestinal protein load is an important source of uremic toxins, and that therapeutic means to reduce this (probiotics, prebiotics, sorbents), if they are not jeopardizing nutritional status, may have added value to the removal obtained by dialysis and should be explored and assessed in controlled trials.

Furthermore, PNA is not only a nutrition term but is also influenced by the metabolic status of the patient. Examination of the body weights during a period starting one month before until one month after the study reveals however that 35% of the patients are stable in weight, while only minor changes (1.8%) were seen in the remaining group with a comparable number of patients with increasing and decreasing body weight, respectively [69.0 (61.3–78.8) kg]. Hence, changes in metabolic status in our patient population did not influence correlations between PNA and uremic toxin concentrations.

A small number of studies suggested that PNA linearly varies with the dialysis dose Kt/V_urea_ [80], [81], but the HEMO study failed to demonstrate any significant change in protein intake attributable to hemodialysis dose [82]. The present study as well did not find any correlation between PNA and Kt/V_urea_ (data not shown).

Strengths of this study are the assessment of a population from one single unit where the same standards of treatment are applied overall, and the inclusion of several parameters with the potential impact on solute concentration. A shortcoming of this study is its transversal nature with only one set of values per patient at one single moment. However, an analysis using average Kt/V_urea_ of the same population over a period of 3 months resulted in the same conclusions (data not shown). In addition, single values of PNA and RRF were used which nevertheless resulted in much higher variances. An analysis using average PNA of the same population over a period of 3 months resulted in even more correlations with uremic toxin concentrations (i.e. also correlated with free IS and free IAA concentrations). Another shortcoming might have been the use of predialysis uremic toxin concentrations. However, an analysis using the time averaged concentration (TAC) of urea, the only solute of which the post dialysis value was available to calculate TAC, gave similar results. Finally, the study results for PNA could have been skewed by not considering the urea concentration after post-dialysis rebound [83] and by only looking at a midweek dialysis session possibly overestimating the weekly value [84] and not including intraweek variability [85]. However, since overestimations by not considering urea rebound are only in the range of 6% [83], since we considered midweek sessions with similar dialysis durations (245±18 min) which were constant during the preceding three months, and since intraweek variations (estimated being 9.3% [85]) are likely to be counterbalanced among patients, our correlation analyses between PNA and uremic toxin concentrations can be considered representative.

## Conclusion

In conclusion, serum concentrations of most uremic toxins are correlated with residual renal function and protein equivalent of nitrogen appearance, and not with dialysis adequacy as assessed by Kt/V_urea_. Hence, we conclude from this observational study that efforts to control intestinal load of uremic toxin precursors by dietary or other interventions, and preserving RRF are important approaches to decrease uremic solute concentration and by extension their toxicity.

## References

[1] Vanholder R, Schepers E, Meert N, Lameire N (2006) What is uremia? Retention versus oxidation. Blood Purif 24: 33–38. 16361838 10.1159/000089434

[2] Vanholder R, Baurmeister U, Brunet P, Cohen G, Glorieux G, et al (2008) A bench to bedside view of uremic toxins. J Am Soc Nephrol 19: 863–870. 18287557 10.1681/ASN.2007121377

[3] Meyer TW, Hostetter TH (2007) Uremia. N Engl J Med 357: 1316–1325. 17898101 10.1056/NEJMra071313

[4] Vanholder R, Davenport A, Hannedouche T, Kooman J, Kribben A, et al (2012) Reimbursement of dialysis: a comparison of seven countries. J Am Soc Nephrol 23: 1291–1298. 22677554 10.1681/ASN.2011111094

[5] Gotch FA, Sargent JA (1985) A mechanistic analysis of the National Cooperative Dialysis Study (NCDS). Kidney Int 28: 526–534. 3934452 10.1038/ki.1985.160

[6] Port FK, Wolfe RA, Hulbert-Shearon TE, McCullough KP, Ashby VB, et al (2004) High dialysis dose is associated with lower mortality among women but not among men. Am J Kidney Dis 43: 1014–1023. 15168381 10.1053/j.ajkd.2004.02.014

[7] Port FK, Pisoni RL, Bragg-Gresham JL, Satayathum SS, Young EW, et al (2004) DOPPS estimates of patient life years attributable to modifiable hemodialysis practices in the United States. Blood Purif 22: 175–180. 14732826 10.1159/000074938

[8] Saran R, Canaud BJ, Depner TA, Keen ML, McCullough KP, et al (2004) Dose of dialysis: key lessons from major observational studies and clinical trials. Am J Kidney Dis 44: 47–53. 15486874 10.1053/j.ajkd.2004.08.011

[9] Sirich TL, Luo FJ, Plummer NS, Hostetter TH, Meyer TW (2012) Selectively increasing the clearance of protein-bound uremic solutes. Nephrol Dial Transplant 27: 1574–1579. 22231033 10.1093/ndt/gfr691 PMC3315673

[10] Basile C, Libutti P, Di Turo AL, Casino FG, Vernaglione L, et al (2011) Removal of uraemic retention solutes in standard bicarbonate haemodialysis and long-hour slow-flow bicarbonate haemodialysis. Nephrol Dial Transplant 26: 1296–1303. 20813765 10.1093/ndt/gfq543

[11] Eloot S, Van Biesen W, Dhondt A, Van de Wynkele H, Glorieux G, et al (2008) Impact of hemodialysis duration on the removal of uremic retention solutes. Kidney Int 73: 765–770. 18160958 10.1038/sj.ki.5002750

[12] Eloot S, Van Biesen W, Dhondt A, De Smet R, Marescau B, et al (2009) Impact of increasing haemodialysis frequency versus haemodialysis duration on removal of urea and guanidino compounds: a kinetic analysis. Nephrol Dial Transplant 24: 2225–2232. 19225018 10.1093/ndt/gfp059

[13] Fagugli RM, Vanholder R, De Smet R, Selvi A, Antolini F, et al (2001) Advanced glycation end products: specific fluorescence changes of pentosidine-like compounds during short daily hemodialysis. Int J Artif Organs 24: 256–262. 11420874

[14] Fagugli RM, De Smet R, Buoncristiani U, Lameire N, Vanholder R (2002) Behavior of non-protein-bound and protein-bound uremic solutes during daily hemodialysis. Am J Kidney Dis 40: 339–347. 12148107 10.1053/ajkd.2002.34518

[15] Rocco MV, Lockridge RS Jr, Beck GJ, Eggers PW, Gassman JJ, et al (2011) The effects of frequent nocturnal home hemodialysis: the Frequent Hemodialysis Network Nocturnal Trial. Kidney Int 80: 1080–1091. 21775973 10.1038/ki.2011.213 PMC3569086

[16] Walsh M, Manns BJ, Klarenbach S, Tonelli M, Hemmelgarn B, et al (2010) The effects of nocturnal compared with conventional hemodialysis on mineral metabolism: A randomized-controlled trial. Hemodial Int 14: 174–181. 20041960 10.1111/j.1542-4758.2009.00418.x

[17] Ayus JC, Mizani MR, Achinger SG, Thadhani R, Go AS, et al (2005) Effects of short daily versus conventional hemodialysis on left ventricular hypertrophy and inflammatory markers: a prospective, controlled study. J Am Soc Nephrol 16: 2778–2788. 16033855 10.1681/ASN.2005040392

[18] Chertow GM, Levin NW, Beck GJ, Depner TA, Eggers PW, et al (2010) In-center hemodialysis six times per week versus three times per week. N Engl J Med 363: 2287–2300. 21091062 10.1056/NEJMoa1001593 PMC3042140

[19] Culleton BF, Walsh M, Klarenbach SW, Mortis G, Scott-Douglas N, et al (2007) Effect of frequent nocturnal hemodialysis vs conventional hemodialysis on left ventricular mass and quality of life: a randomized controlled trial. JAMA 298: 1291–1299. 17878421 10.1001/jama.298.11.1291

[20] McGregor DO, Buttimore AL, Lynn KL, Nicholls MG, Jardine DL (2001) A Comparative Study of Blood Pressure Control with Short In-Center versus Long Home Hemodialysis. Blood Purif 19: 293–300. 11244189 10.1159/000046957

[21] Meyer TW, Sirich TL, Hostetter TH (2011) Dialysis cannot be dosed. Semin Dial 24: 471–479. 21929590 10.1111/j.1525-139X.2011.00979.x PMC4124940

[22] Locatelli F, Martin-Malo A, Hannedouche T, Loureiro A, Papadimitriou M, et al (2009) Effect of membrane permeability on survival of hemodialysis patients. J Am Soc Nephrol 20: 645–654. 19092122 10.1681/ASN.2008060590 PMC2653681

[23] Santoro A, Mancini E, Bolzani R, Boggi R, Cagnoli L, et al (2008) The effect of on-line high-flux hemofiltration versus low-flux hemodialysis on mortality in chronic kidney failure: a small randomized controlled trial. Am J Kidney Dis 52: 507–518. 18617304 10.1053/j.ajkd.2008.05.011

[24] Duranton F, Cohen G, De Smet R, Rodriguez M, Jankowski J, et al (2012) Normal and Pathologic Concentrations of Uremic Toxins. J Am Soc Nephrol 23: 1258–1270. 22626821 10.1681/ASN.2011121175 PMC3380651

[25] Vanholder R, De Smet R, Glorieux G, Argiles A, Baurmeister U, et al (2003) Review on uremic toxins: classification, concentration, and interindividual variability. Kidney Int 63: 1934–1943. 12675874 10.1046/j.1523-1755.2003.00924.x

[26] Eloot S, Schepers E, Barreto DV, Barreto FC, Liabeuf S, et al (2011) Estimated glomerular filtration rate is a poor predictor of concentration for a broad range of uremic toxins. Clin J Am Soc Nephrol 6: 1266–1273. 21617084 10.2215/CJN.09981110 PMC3109921

[27] Neirynck N, Eloot S, Glorieux G, Barreto DV, Barreto FC, et al (2012) Estimated glomerular filtration rate is a poor predictor of the concentration of middle molecular weight uremic solutes in chronic kidney disease. PloS One 7: e44201. 22952928 10.1371/journal.pone.0044201 PMC3432070

[28] Vanholder R, Eloot S, Schepers E, Neirynck N, Glorieux G, et al (2011) An Obituary for GFR as the Main Marker for Kidney Function? Semin Dial 25: 9–14. 22141430 10.1111/j.1525-139X.2011.01003.x

[29] Schepers E, Glorieux G, Vanholder R (2010) The gut: the forgotten organ in uremia? Blood Purif 29: 130–136. 20093818 10.1159/000245639

[30] Daugirdas JT (1993) Second generation logarithmic estimates of single-pool variable volume Kt/V: an analysis of error. J Am Soc Nephrol 4: 1205–1213. 8305648 10.1681/ASN.V451205

[31] Section II (2002) Haemodialysis adequacy (2002) Nephrol Dial Transplant 17 Suppl 7: 16–31. 12386211

[32] Vanholder R, Hoefliger N, de SR, Ringoir S (1992) Extraction of protein bound ligands from azotemic sera: comparison of 12 deproteinization methods. Kidney Int 41: 1707–1712. 1501426 10.1038/ki.1992.244

[33] Daugirdas JT, Depner TA, Greene T, Levin NW, Chertow GM, et al (2010) Standard Kt/Vurea: a method of calculation that includes effects of fluid removal and residual kidney clearance. Kidney Int 77: 637–644. 20107428 10.1038/ki.2009.525

[34] Daugirdas JT, Smye SW (1997) Effect of a two compartment distribution on apparent urea distribution volume. Kidney Int 51: 1270–1273. 9083296 10.1038/ki.1997.173

[35] Tattersall JE, DeTakats D, Chamney P, Greenwood RN, Farrington K (1996) The post-hemodialysis rebound: predicting and quantifying its effect on Kt/V. Kidney Int 50: 2094–2102. 8943495 10.1038/ki.1996.534

[36] Section I (2002) Measurement of renal function, when to refer and when to start dialysis (2002) Nephrol Dial Transplant 17 Suppl 7: 7–15. 10.1093/ndt/17.suppl_7.7 12386205

[37] National Kidney Foundation (2000) NKF KDOQI Guidelines: Nutrition in Chronic Renal Failure. Am J Kidney Dis 35: S1–S140. 10895784 10.1053/ajkd.2000.v35.aajkd03517

[38] Fouque D, Vennegoor M, ter WP, Wanner C, Basci A, et al (2007) EBPG guideline on nutrition. Nephrol Dial Transplant 22 Suppl 2: ii45–ii87. 17507426 10.1093/ndt/gfm020

[39] Bammens B, Evenepoel P, Keuleers H, Verbeke K, Vanrenterghem Y (2006) Free serum concentrations of the protein-bound retention solute p-cresol predict mortality in hemodialysis patients. Kidney Int 69: 1081–1087. 16421516 10.1038/sj.ki.5000115

[40] Barreto FC, Barreto DV, Liabeuf S, Meert N, Glorieux G, et al (2009) Serum indoxyl sulfate is associated with vascular disease and mortality in chronic kidney disease patients. Clin J Am Soc Nephrol 4: 1551–1558. 19696217 10.2215/CJN.03980609 PMC2758258

[41] Kielstein JT, Impraim B, Simmel S, Bode-Boger SM, Tsikas D, et al (2004) Cardiovascular effects of systemic nitric oxide synthase inhibition with asymmetrical dimethylarginine in humans. Circulation 109: 172–177. 14662708 10.1161/01.CIR.0000105764.22626.B1

[42] Liabeuf S, Barreto DV, Barreto FC, Meert N, Glorieux G, et al (2010) Free p-cresylsulphate is a predictor of mortality in patients at different stages of chronic kidney disease. Nephrol Dial Transplant 25: 1183–1191. 19914995 10.1093/ndt/gfp592

[43] Meijers BK, Bammens B, De Moor B, Verbeke K, Vanrenterghem Y, et al (2008) Free p-cresol is associated with cardiovascular disease in hemodialysis patients. Kidney Int 73: 1174–1180. 18305466 10.1038/ki.2008.31

[44] Meijers BK, Van Kerckhoven S, Verbeke K, Dehaen W, Vanrenterghem Y, et al (2009) The uremic retention solute p-cresyl sulfate and markers of endothelial damage. Am J Kidney Dis 54: 891–901. 19615803 10.1053/j.ajkd.2009.04.022

[45] Yilmaz MI, Sonmez A, Saglam M, Qureshi AR, Carrero JJ, et al (2008) ADMA levels correlate with proteinuria, secondary amyloidosis, and endothelial dysfunction. J Am Soc Nephrol 19: 388–395. 18199801 10.1681/ASN.2007040461 PMC2396733

[46] Yu MA, Sanchez-Lozada LG, Johnson RJ, Kang DH (2010) Oxidative stress with an activation of the renin-angiotensin system in human vascular endothelial cells as a novel mechanism of uric acid-induced endothelial dysfunction. J Hypertens 28: 1234–1242. 20486275

[47] Zoccali C, Maio R, Mallamaci F, Sesti G, Perticone F (2006) Uric acid and endothelial dysfunction in essential hypertension. J Am Soc Nephrol 17: 1466–1471. 16611716 10.1681/ASN.2005090949

[48] Cheung AK, Rocco MV, Yan G, Leypoldt JK, Levin NW, et al (2006) Serum beta-2 microglobulin levels predict mortality in dialysis patients: results of the HEMO study. J Am Soc Nephrol 17: 546–555. 16382021 10.1681/ASN.2005020132

[49] Cheung AK, Greene T, Leypoldt JK, Yan G, Allon M, et al (2008) Association between serum 2-microglobulin level and infectious mortality in hemodialysis patients. Clin J Am Soc Nephrol 3: 69–77. 18057309 10.2215/CJN.02340607 PMC2390979

[50] Eloot S, Torremans A, De Smet R, Marescau B, De Wachter D, et al (2005) Kinetic behavior of urea is different from that of other water-soluble compounds: the case of the guanidino compounds. Kidney Int 67: 1566–1575. 15780113 10.1111/j.1523-1755.2005.00238.x

[51] Eloot S, Torremans A, De Smet R, Marescau B, De Deyn PP, et al (2007) Complex compartmental behavior of small water-soluble uremic retention solutes: evaluation by direct measurements in plasma and erythrocytes. Am J Kidney Dis 50: 279–288. 17660029 10.1053/j.ajkd.2007.05.009

[52] Boumendil-Podevin EF, Podevin RA, Richet G (1975) Uricosuric agents in uremic sera. Identification of indoxyl sulfate and hippuric acid. J Clin Invest 55: 1142–1152. 1133164 10.1172/JCI108031 PMC301867

[53] Spoto B, Parlongo RM, Parlongo G, Sgro' E, Zoccali C (2007) The enzymatic machinery for ADMA synthesis and degradation is fully expressed in human adipocytes. J Nephrol 20: 554–559. 17918140

[54] Vaziri ND, Freel RW, Hatch M (1995) Effect of chronic experimental renal insufficiency on urate metabolism. J Am Soc Nephrol 6: 1313–1317. 8589304 10.1681/ASN.V641313

[55] Guarner F, Malagelada JR (2003) Gut flora in health and disease. Lancet 361: 512–519. 12583961 10.1016/S0140-6736(03)12489-0

[56] Aronov PA, Luo FJ, Plummer NS, Quan Z, Holmes S, et al (2011) Colonic contribution to uremic solutes. J Am Soc Nephrol 22: 1769–1776. 21784895 10.1681/ASN.2010121220 PMC3171947

[57] Kusumoto M, Kamobayashi H, Sato D, Komori M, Yoshimura M, et al (2011) Alleviation of cisplatin-induced acute kidney injury using phytochemical polyphenols is accompanied by reduced accumulation of indoxyl sulfate in rats. Clin Exp Nephrol 15: 820–830. 21858734 10.1007/s10157-011-0524-z

[58] Swann JR, Tuohy KM, Lindfors P, Brown DT, Gibson GR, et al (2011) Variation in antibiotic-induced microbial recolonization impacts on the host metabolic phenotypes of rats. J Proteome Res 10: 3590–3603. 21591676 10.1021/pr200243t

[59] Fujita T, Ishihara K, Yasuda S, Nakamura T, Maeda M, et al (2012) In vivo kinetics of indoxyl sulfate in humans and its renal interaction with angiotensin-converting enzyme inhibitor quinapril in rats. J Pharmacol Exp Ther 341: 626–633. 22389425 10.1124/jpet.111.187732

[60] Heinzmann SS, Merrifield CA, Rezzi S, Kochhar S, Lindon JC, et al (2012) Stability and robustness of human metabolic phenotypes in response to sequential food challenges. J Proteome Res 11: 643–655. 21999107 10.1021/pr2005764

[61] Krediet RT (2006) How to preserve residual renal function in patients with chronic kidney disease and on dialysis? Nephrol Dial Transplant 21 Suppl 2: ii42–ii46. 16825260 10.1093/ndt/gfl137

[62] Suda T, Hiroshige K, Ohta T, Watanabe Y, Iwamoto M, et al (2000) The contribution of residual renal function to overall nutritional status in chronic haemodialysis patients. Nephrol Dial Transplant 15: 396–401. 10692527 10.1093/ndt/15.3.396

[63] Evenepoel P, Bammens B, Verbeke K, Vanrenterghem Y (2006) Superior dialytic clearance of beta(2)-microglobulin and p-cresol by high-flux hemodialysis as compared to peritoneal dialysis. Kidney Int 70: 794–799. 16820785 10.1038/sj.ki.5001640

[64] Fry AC, Singh DK, Chandna SM, Farrington K (2007) Relative importance of residual renal function and convection in determining beta-2-microglobulin levels in high-flux haemodialysis and on-line haemodiafiltration. Blood Purif 25: 295–302. 17622712 10.1159/000104870

[65] Penne EL, van der Weerd NC, Blankestijn PJ, van den Dorpel MA, Grooteman MP, et al (2010) Role of residual kidney function and convective volume on change in beta2-microglobulin levels in hemodiafiltration patients. Clin J Am Soc Nephrol 5: 80–86. 19965537 10.2215/CJN.03340509 PMC2801642

[66] Bammens B, Evenepoel P, Verbeke K, Vanrenterghem Y (2003) Removal of middle molecules and protein-bound solutes by peritoneal dialysis and relation with uremic symptoms. Kidney Int 64: 2238–2243. 14633148 10.1046/j.1523-1755.2003.00310.x

[67] Marquez IO, Tambra S, Luo FY, Li Y, Plummer NS, et al (2011) Contribution of residual function to removal of protein-bound solutes in hemodialysis. Clin J Am Soc Nephrol 6: 290–296. 21030575 10.2215/CJN.06100710 PMC3052218

[68] Viaene L, Bammens B, Meijers BK, Vanrenterghem Y, Vanderschueren D, et al (2012) Residual renal function is an independent determinant of serum FGF-23 levels in dialysis patients. Nephrol Dial Transplant 27: 2017–2022. 22025115 10.1093/ndt/gfr596

[69] Schwedler SB, Metzger T, Schinzel R, Wanner C (2002) Advanced glycation end products and mortality in hemodialysis patients. Kidney Int 62: 301–310. 12081592 10.1046/j.1523-1755.2002.00423.x

[70] Kalantar-Zadeh K, Block G, Humphreys MH, McAllister CJ, Kopple JD (2004) A low, rather than a high, total plasma homocysteine is an indicator of poor outcome in hemodialysis patients. J Am Soc Nephrol 15: 442–453. 14747392 10.1097/01.asn.0000107564.60018.51

[71] Suliman M, Stenvinkel P, Qureshi AR, Kalantar-Zadeh K, Barany P, et al (2007) The reverse epidemiology of plasma total homocysteine as a mortality risk factor is related to the impact of wasting and inflammation. Nephrol Dial Transplant 22: 209–217. 16982634 10.1093/ndt/gfl510

[72] Suliman ME, Stenvinkel P, Qureshi AR, Barany P, Heimburger O, et al (2004) Hyperhomocysteinemia in relation to plasma free amino acids, biomarkers of inflammation and mortality in patients with chronic kidney disease starting dialysis therapy. Am J Kidney Dis 44: 455–465. 15332218

[73] Bammens B, Evenepoel P, Verbeke K, Vanrenterghem Y (2004) Impairment of small intestinal protein assimilation in patients with end-stage renal disease: extending the malnutrition-inflammation-atherosclerosis concept. Am J Clin Nutr 80: 1536–1543. 15585765 10.1093/ajcn/80.6.1536

[74] Evenepoel P, Meijers BK, Bammens BR, Verbeke K (2009) Uremic toxins originating from colonic microbial metabolism. Kidney Int Suppl S12–S19. 19946322 10.1038/ki.2009.402

[75] Schulman G (2004) The dose of dialysis in hemodialysis patients: impact on nutrition. Semin Dial 17: 479–488. 15660579 10.1111/j.0894-0959.2004.17609.x

[76] Marcen R, Teruel JL, de la Cal MA, Gamez C (1997) The impact of malnutrition in morbidity and mortality in stable haemodialysis patients. Spanish Cooperative Study of Nutrition in Hemodialysis. Nephrol Dial Transplant 12: 2324–2331. 9394319 10.1093/ndt/12.11.2324

[77] Bergstrom J (1995) Nutrition and mortality in hemodialysis. J Am Soc Nephrol 6: 1329–1341. 8589306 10.1681/ASN.V651329

[78] Acchiardo SR, Moore LW, Latour PA (1983) Malnutrition as the main factor in morbidity and mortality of hemodialysis patients. Kidney Int Suppl 16: S199–S203. 6429404

[79] Laird NM, Berkey CS, Lowrie EG (1983) Modeling success or failure of dialysis therapy: the National Cooperative Dialysis Study. Kidney Int Suppl S101–S106. 6345890

[80] Lindsay RM, Spanner E (1989) A hypothesis: the protein catabolic rate is dependent upon the type and amount of treatment in dialyzed uremic patients. Am J Kidney Dis 13: 382–389. 2719025 10.1016/s0272-6386(89)80021-6

[81] Azar AT, Wahba K, Mohamed AS, Massoud WA (2007) Association between dialysis dose improvement and nutritional status among hemodialysis patients. Am J Nephrol 27: 113–119. 17308372 10.1159/000099836

[82] Rocco MV, Dwyer JT, Larive B, Greene T, Cockram DB, et al (2004) The effect of dialysis dose and membrane flux on nutritional parameters in hemodialysis patients: results of the HEMO Study. Kidney Int 65: 2321–2334. 15149346 10.1111/j.1523-1755.2004.00647.x

[83] Stegeman CA, Huisman RM, de RB, Joostema A, de Jong PE (1995) Determination of protein catabolic rate in patients on chronic intermittent hemodialysis: urea output measurements compared with dietary protein intake and with calculation of urea generation rate. Am J Kidney Dis 25: 887–895. 7771485 10.1016/0272-6386(95)90571-5

[84] Lopot F, Kotyk P, Blaha J, Valek A (1995) Analysis of the urea generation rate and the protein catabolic rate in hemodialyzed patients. Artif Organs 19: 832–836. 8573004 10.1111/j.1525-1594.1995.tb02436.x

[85] Kloppenburg WD, Stegeman CA, Hooyschuur M, van der Ven J, de Jong PE, et al (1999) Assessing dialysis adequacy and dietary intake in the individual hemodialysis patient. Kidney Int 55: 1961–1969. 10231460 10.1046/j.1523-1755.1999.00412.x

